# CD63 + tumor-associated macrophages drive the progression of hepatocellular carcinoma through the induction of epithelial-mesenchymal transition and lipid reprogramming

**DOI:** 10.1186/s12885-024-12472-7

**Published:** 2024-06-07

**Authors:** Shiqi Liu, Shuairan Zhang, Hang Dong, Xiuli Jin, Jing Sun, Haonan Zhou, Yifan Jin, Yiling Li, Gang Wu

**Affiliations:** 1 Department of Hepatobiliary Surgery, The First Affiliated Hospital of China Medical University, Shenyang, People’s Republic of China; 2 Department of Gastroenterology, The First Affiliated Hospital of China Medical University, Shenyang, People’s Republic of China; 3 Phase I Clinical Trails Center, The People’s Hospital of China Medical University, Shenyang, People’s Republic of China

**Keywords:** Hepatocellular carcinoma, CD63, Tumor-associated macrophages

## Abstract

**Background:**

Tumor-associated macrophages (TAMs) constitute a substantial part of human hepatocellular carcinoma (HCC). The present study was devised to explore TAM diversity and their roles in HCC progression.

**Methods:**

Through the integration of multiple 10 × single-cell transcriptomic data derived from HCC samples and the use of consensus nonnegative matrix factorization (an unsupervised clustering algorithm), TAM molecular subtypes and expression programs were evaluated in detail. The roles played by these TAM subtypes in HCC were further probed through pseudotime, enrichment, and intercellular communication analyses. Lastly, vitro experiments were performed to validate the relationship between CD63, which is an inflammatory TAM expression program marker, and tumor cell lines.

**Results:**

We found that the inflammatory expression program in TAMs had a more obvious interaction with HCC cells, and CD63, as a marker gene of the inflammatory expression program, was associated with poor prognosis of HCC patients. Both bulk RNA-seq and vitro experiments confirmed that higher TAM CD63 expression was associated with the growth of HCC cells as well as their epithelial-mesenchymal transition, metastasis, invasion, and the reprogramming of lipid metabolism.

**Conclusions:**

These analyses revealed that the TAM inflammatory expression program in HCC is closely associated with malignant tumor cells, with the hub gene CD63 thus representing an ideal target for therapeutic intervention in this cancer type.

**Supplementary Information:**

The online version contains supplementary material available at 10.1186/s12885-024-12472-7.

## Background

Hepatocellular carcinoma (HCC) poses a significant global health challenge due to its heterogeneity and limited treatment options [1]. Even with surgical treatment as an option for some patients, the high rates of recurrence and poor prognostic outcomes experienced by patients with unresectable diseases underscore the pressing need to establish novel interventions [2]. While the tumor microenvironment (TME) consists of many cell types, tumor-associated macrophages (TAMs), in particular, have been a focus of substantial interest regarding the roles that they play in the progression of HCC [3].

TAMs exhibit a high degree of plasticity, contributing to metastatic tumor progression, immune evasion, angiogenic activity, and resistance to therapy via the secretion of a range of factors including chemokines, cytokines, and matrix metalloproteinases (MMPs) [4–7]. Efforts to target TAMs have been advanced as an attractive form of cancer immunotherapy, but the persistent lack of reliable and specific markers associated with TAMs has hampered these efforts [8, 9].

The epithelial-mesenchymal transition (EMT) process is integral to the invasive and metastatic progression of HCC. When tumor cells undergo the EMT, they present with greater migratory and invasive activity and altered adhesion molecule expression [10, 11]. A growing body of evidence suggests that the dysregulation of cholesterol and lipid metabolism is involved in tumor initiation and progression [12]. Lipids are a vital source of energy that can fuel metastatic progression [13, 14]. Lipids and cholesterol can conjoin on cell membranes, forming lipid rafts, and assemble alongside numerous receptors, ligands, and iron channel proteins, acting as functional units [15].

Here, an effort was made to better understand TAM heterogeneity in HCC through the integration of single-cell RNA sequencing (scRNA-seq) data. The specific goal of these analyses was to better characterize communication networks linking TAMs and tumor cells, and to clarify the impact of these interactions on the survival of patients. These analyses revealed that high CD63 expression in the inflammatory TAM expression program was correlated with aggressive HCC phenotypes. Vitro experiments also confirmed these results, with macrophage-derived CD63 serving to promote HCC cell proliferative, invasive, and metastatic activity. CD63 + TAMs were also found to promote EMT induction and dysregulated lipid metabolism within HCC cells. Together, these results emphasize the potential value of CD63 as a novel target for the treatment of HCC, given that its functional role in TAMs can drive malignant disease progression.

Together, these findings underscore the necessity to explore TAM heterogeneity while also establishing CD63 as an important driver of HCC progression. Efforts to target CD63 in TAMs may represent a promising approach to the development of effective treatments for HCC.

## Methods

### Data retrieval and processing

Publically available HCC scRNA-seq datasets were initially identified through a systematic search effort, leading to the identification of two datasets generated with the 10 × Genomics single-cell 3’-sequencing platform, including one from the National Institutes of Health (NIH) and one from the Beijing Proteome Research Center (BPRC) [16, 17]. To validate the present findings on a larger scale, two large-scale bulk RNA-seq cohorts pertaining to HCC were also integrated. The log2(count + 1) processed bulk RNA-seq data (*n* = 424) and clinical details corresponding to liver HCC (LIHC) patients were accessed through The Cancer Genome Atlas (TCGA) using the UCSC Xena browser (https://xenabrowser.net/datapages/). Gene expression and clinical data for 241 patients from a Japanese cohort (*n* = 241) were also accessed via the International Cancer Genome Consortium (ICGC).

R (v4.1.2) was used to conduct scRNA-seq analyses with the Seurat package (v4.0.2). Initially, data were converted into Seurat objects, with the following quality control steps: (1) only HCC tissue-derived cells were retained for analysis; (2) only cells with 200–6000 detected genes were retained for analysis; (3) genes were excluded if expressed in fewer than 5 cells; and (4) cells with > 10% detected mitochondrial genes were excluded. Those cells with fewer than 200 genes were regarded as dead or broken cells, whereas the detection of > 6000 genes was considered indicative of doublets resulting from droplet encapsulation errors. The Seurat 'NormalizeData' and 'ScaleData' functions were used for log normalization and linear regression analyses to generate gene expression matrices. The top 2000 highly variable genes (HVGs) were selected with the 'FindVariableFeatures' function. Dimensionality reduction was achieved through a principal component analysis (PCA) approach, with the uniform manifold approximation and projection (UMAP) approach being utilized for cell visualization. The 'FindClusters' function was employed at a resolution of 0.4 to cluster cells, and ‘SingleR’ was used to annotate cell types based on classic markers. Correction for batch effects between samples and cohorts was achieved using the ‘Harmony’ algorithm [18].

### Differential expression and functional enrichment analyses

Differential analysis of gene expression was performed using two algorithms: the 'edgeR' algorithm or the Wilcoxon signed rank test [19]. Differentially expressed genes (DEGs) were selected using the default parameters of the Wilcoxon likelihood ratio test and the 'FindMarkers' function in Seurat. DEGs were required to have at least 10% expression in cells and an average log2(fold change) greater than 0.25. Enrichment analysis for Gene Ontology (GO) and Kyoto Encyclopedia of Genes and Genomes (KEGG) was conducted using the 'clusterProfiler' package. Significance was determined by an adjusted p value less than 0.05. To calculate the enrichment score of gene sets in each cell, the 'AddModuleScore' function was utilized.

### Expression programs analyses

A consensus nonnegative matrix factorization (cNMF) algorithm was leveraged for the identification of transcriptomic expression programs. The program signature consisted of the top 30 genes with the highest program weight. Subsequently, the scores for each expression program in every cell were computed based on this signature [20, 21].

### Gene regulatory network analyses

The Single-Cell rEgulatory Network Inference and Clustering (SCENIC) algorithm was employed to identify regulons [22], which consist of transcription factors and associated gene targets. To assess regulon activity, the ‘AUCell’ package was employed for area under the curve (AUC) calculations, and the entropy-based regulon specificity score (RSS) was used to evaluate regulon occupancy [23]. Hub regulons were established as the top 10 regulators exhibiting the highest RSS in each of the cellular subtypes.

### Trajectory analyses

To investigate the differentiation/transformation of TAMs in the HCC microenvironment, pseudotime trajectory analysis was performed using Monocle2 [24]. The top 2000 highly variable genes (HVGs) were selected for constructing trajectories, whereby dimensionality reduction was achieved using the 'DDRTree' function. Dimensionality reduction plots and pseudotime heatmaps were visualized using the "plot_cell_trajectory" and "plot_pseudotime_heatmap" functions, respectively.

### Cell‒cell interaction (CCI) analyses

To predict interactions between different cell types, we employed CellPhoneDB (v2.1.0), which is based on known ligand‒receptor pairs[25]. In total, 1000 permutations were used to calculate the null distribution of mean ligand-receptor pair expression in each random cell. Cutoff values based on the mean log gene expression distribution for all genes in each type of cell were used to determine expression thresholds for particular receptors or ligands, with a mean expression > 0.1 and a *P* < 0.05 being considered indicative of significant interactions.

### Cell culture

The human monocytic cell line THP-1 and the liver cancer cell lines HepG2 and Huh7 were purchased from the Shanghai Cell Bank (Shanghai, China). THP-1-derived macrophages were produced by stimulating THP-1 cells using PMA (100 ng/mL, Sigma, P1585) for 48 h, with the resultant cells being cultured in RPMI-1640 containing 10% FBS and 1% penicillin/streptomycin. Huh7 and HepG2 cells were cultured in DMEM (Hyclone) with 10% FBS and 1% penicillin/streptomycin. All cells were cultured in 37 °C 5% CO_2_ incubators (Thermo, MA, USA).

### Lentivirus transfection

Lentiviruses carrying CD63 knockdown or control vectors were obtained from Gene-Chem and used to transfected THP-1 cells as directed, with > 80% of cells being GFP positive being considered indicative of successful transfection. Following lentiviral transduction, puromycin (3 µg/ml) was used to select cells for 15 days, after which this puromycin concentration was maintained at 1 µg/ml. CD63 overexpression was achieved by transfecting tumor cells at 60–70% confluence with a CD63 plasmid (Gene-Pharma Technologies, Shanghai, China). Utilized shRNA and plasmid sequences are presented in Supplementary Material 2, Table S1.

### Co-culture

A co-culture system was generated with 6-well transwells (3450, Corning). Initially, THP-1-derived macrophages in which CD63 was overexpressed or knocked down were added into the upper chamber, followed by the transfer of these seeded inserts into 6-well plates pre-seeded with Huh7 or HepG2 cells. After an additional 48 h co-culture period, liver cancer cells from the lower chamber were harvested for functional analyses.

### RNA extraction and quantitative real-time PCR (qRT‒PCR)

TRIzol (Invitrogen, USA) was used to isolate cellular total RNA as directed, after which the PrimeScript RT reagent Kit (Takara) was used to produce cDNA. Levels of CD63 mRNA expression were assessed using TB Green® (RR820A, Takara) with a Light Cycler 480 II Real-Time PCR system (Roche Diagnostics). The primers used were designed and synthesized by Sangon Biotech. The primer sequences of relevant genes can be found in Supplementary Material 2, Table S1.

### Protein extraction and western blotting

Protein lysis buffer (KeyGEN BioTECH) was used for the extraction of total cellular proteins which were quantified with a BCA kit as directed. Then, SDS-PAGE was used to separate 20 µg of protein extract per sample and these were transferred onto PVDF membranes (Millipore, MA, USA). After blocking blots for 2 h at room temperature using 5% skim milk, they were probed with primary antibodies overnight at 4 °C, rinsed thrice with TBST (15 min/wash), incubated for 2 h at room temperature with secondary antibodies, rinsed with TBST, and proteins were detected with an electrochemiluminescence (ECL) detection kit (Thermol Biotech, IL, USA). Antibody brands and corresponding numbers are listed in Supplementary Material 2, Table S1.

### Cell viability assays

CCK8**(**Cell Counting Kit-8), EdU, and colony formation assays were employed as measures of cellular viability. For CCK8 assays, cells were added to 96-well plates. Absorbance at 450 nm in each well was assessed with a microplate reader (SpectraMax Absorbance Reader, USA). For EdU assays, cells were added to 12-well plates for 24 h at 30–40% confluency, followed by incubation for 2 h in EdU-containing media with a BeyoClick™EdU kit (C0071S) as directed. These cells were imaged with a fluorescence microscope (Leica DMi8, THUNDER Imager, Germany). Colony formation assays were performed by seeding 6-well plates with 1 × 10^3^ cells for 14 days, and then fixing the resultant colonies with methanol, staining them with crystal violet, and quantifying them with Image J.

### Transwell assays

In migration assays, 2 × 10^4^ cells suspended in 200 µl of high-glucose serum-free DMEM were placed in the upper chamber of a Transwell (Costar, USA), whereas 600 µl of DMEM containing 20% FBS was added to the lower chamber. Invasion assays were conducted with this same general approach, except that the Transwell chamber had been precoated for 6 h with 50 μl of Matrigel (1:9, BD Bioscience, USA). Following a 48 h incubation in a tissue culture incubator, a cotton swab was utilized to remove those cells inside the chamber, while the cells on the basement membrane of the chamber were stained using hematoxylin and eosin, fixed using neutral resin, and imaged with a Leica DM3000 microscope (Leica, Wetzlar, Germany). Cells were then counted with Image-Pro 6.0.

### Apoptosis and cell cycle analyses

An APC and PI apoptosis kit (KGA105, KeyGEN BioTECH) and a cell cycle kit (KGA512, KeyGEN BioTECH) were respectively used as directed as a means of assessing cellular apoptosis and cell cycle progression, followed by flow cytometry analyses.

### Lipid content assays

To detect neutral lipids within cells, the BODIPY 493/503 fluorescent dye was used as directed, adding this dye to the culture medium followed by a 30 min incubation at 37 °C. Cells were subsequently fixed using 4% paraformaldehyde, and a multichannel fluorescence microscope (Leica DMi8, THUNDER Imager, Germany) was employed to image neutral lipid staining results. Differences in fluorescence intensity among groups were assessed. Tumor cells were lysed for 30 min with lysis buffer at 4 °C, followed by the use of chloroform/methanol (2:1) for lipid extraction. Cellular free fatty acid (FFA) and cholesterol (CL) levels were then analyzed with the EnzyChrom™ FFA and CL kits (Bioassay Systems), as directed.

### Statistical analysis

All experiments were conducted at least three times, and results are given as means ± SD. R software (version 4.2.1) and GraphPad Prism 8.0 were used to conduct statistical analyses. Data were analyzed with Student’s t-tests or one-way ANOVAs as appropriate, with spearman analyses being used to assess correlations between the expression of gene pairs. Kaplan–Meier curves were employed to estimate survival probabilities, and the R ‘survminer’ package was used to select the optimal survival cutoff based on the maximally selected rank statistic. A two-sided test with a significance level of *P* < 0.05 was considered statistically significant.

## Results

### Characterization of the HCC cellular landscape with single-cell resolution

**Fig. 1:**
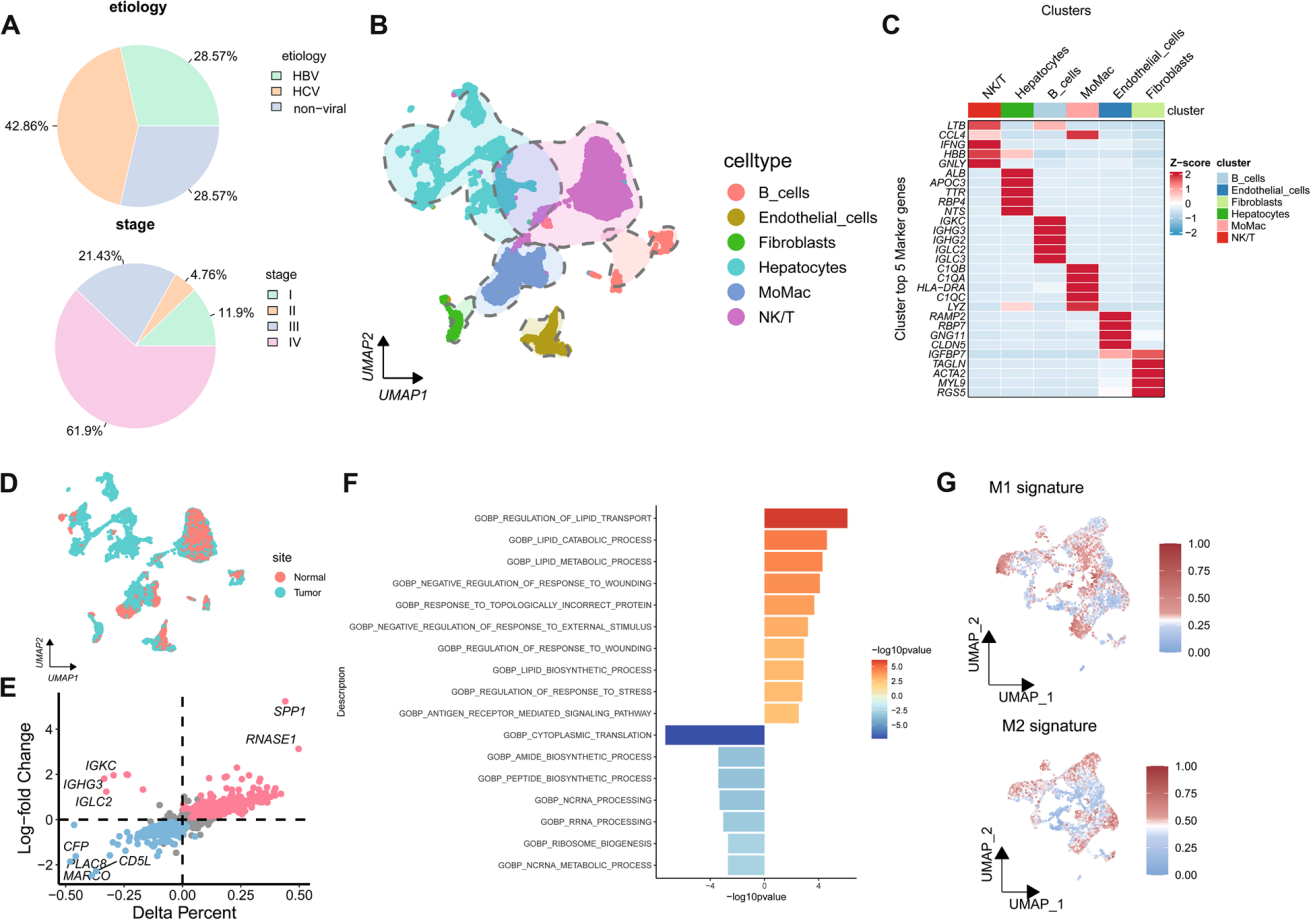
Cellular Landscape and Functional Heterogeneity of TAMs in HCC. **A **Distribution of Patients with Different Etiologies and AJCC Stages in the scRNA-seq Dataset. **B **Visualization of All 104,428 Cells Using the Uniform Manifold Approximation and Projection (UMAP) Technique, with Cells Colored by Cell Type. **C **Heatmap displaying the top 5 marker genes for each cell type. A Random Subset of 100 Cells from Each Cell Type was Selected for Visualization. **D **Visualization of All 104,428 Cells Using the UMAP Technique, with Cells Colored by Normal or Tumor Tissue. **E **Scatter Plot Showing Differentially Expressed Genes Between Monocytes/Macrophages in Normal Tissue and TAMs in Tumor Tissue. Each dot represents a gene, with red dots indicating upregulated genes and blue dots indicating downregulated genes. **F **Bar plot illustrating the Gene Ontology functional enrichment analysis of TAMs in HCC. Red represents upregulated functions, and blue represents downregulated functions. **G **Calculation of M1 and M2 signatures for TAMs Using AUCell

In total, 104,428 cells passed the quality control standards for inclusion in this analysis, of which the NIH and BRPC cohorts respectively contributed 43,011 and 61,417 cells. HCV-related HCC samples comprised the greatest proportion (42.86%) of the scRNA-seq data, whereas HBV-related HCC and nonviral-related HCC samples each comprised 28.57% of these data. Among the HCC samples, 61.9% were classified as stage IV, with stages III, II, and I representing 21.43%, 4.76%, and 11.9% of the samples, respectively (Fig. 1A). Next, a comprehensive single-cell transcriptomic atlas of the HCC TME was established that included B cells, endothelial cells, fibroblasts, epithelial cells, monocytes/macrophages, and NK/T cells (Fig. 1B). The top 5 genes with the most significant expression in each of these cell types are shown in Fig. 1C.

Strikingly, monocytes/macrophages presented with distribution patterns that were distinct when comparing normal and malignant tissues of different types, supporting a role for these cells in the pathogenesis of HCC (Fig. 1D). A subsequent analysis of DEGs expressed in monocytes/macrophages from normal and tumor tissues was conducted, revealing 80 upregulated DEGs in monocytes/macrophages and 150 downregulated DEGs in TAMs (Fig. 1E,Supplementary Material 2, Table S2). Gene Ontology (GO) enrichment analyses indicated that the DEGs upregulated in TAMs were primarily related to lipid metabolism, stress response, and antigen response pathways (Fig. 1F, Supplementary Material 2, Table S3). TAMs are frequently classified into the M1 and M2 phenotypes. The evaluation of these cells for classical M1 and M2 markers was thus performed (Supplementary Material 2, Table S4), revealing significant differentiation of certain HCC-associated TAM populations according to these M1/M2 phenotypes, although certain clusters of these cells were not effectively classified based on these M1/M2 markers (Fig. 1G).

### Assessment of transcriptome heterogeneity of TAMs in HCC

**Fig. 2:**
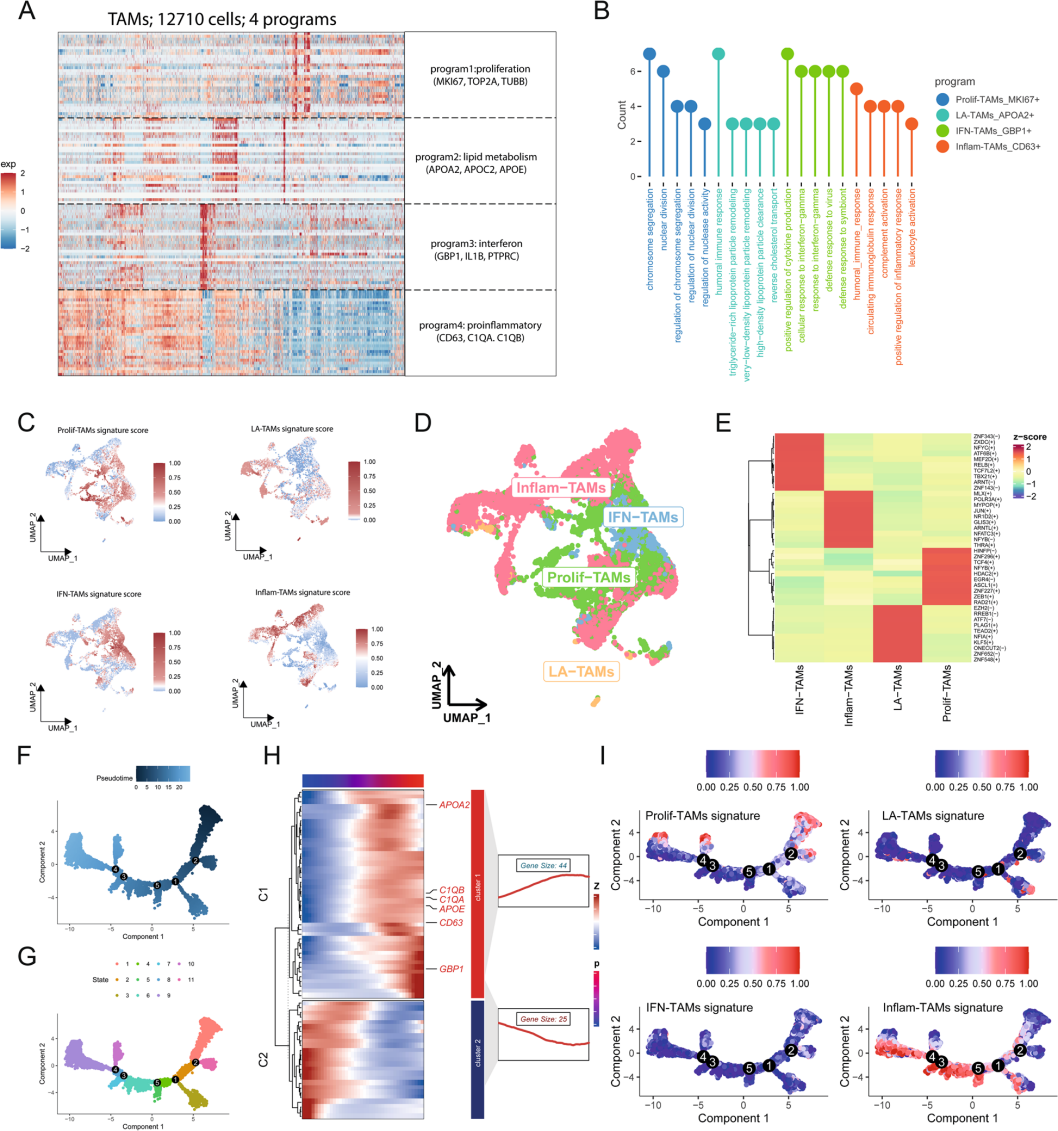
Deciphering Expression Programs Reveals Functional Features of TAMs. **A **Consensus nonnegative matrix factorization (cNMF) analysis of TAMs. The Heatmap Presents Representative Markers of Transcriptome Modules Derived from TAMs, with the Main Functions and Top 3 Ranked Genes of Each Module Shown on the Right. **B **Identification of the top 5 enriched gene sets from representative markers of transcriptome modules derived from TAMs. **C **Calculation of Module Scores for TAMs Using AUCell based on the cNMF Algorithm. **D **Visualization of the Four Distinct TAM Subtypes Based on Their Module Scores. **E **Heatmap of the Top 10 Ranked Regulons in TAM Subtypes. **F**, **G **Potential Developmental Trajectory of TAMs Inferred through the Monocle 2 Algorithm. **H **Identification of differentially expressed genes over pseudotime. **I **Trajectory of Module Scores Along the Pseudotime Defined by the Monocle 2 Algorithm

To begin exploring HCC-related TAM expression patterns, distinct expression modules were elucidated with a cNMF approach. TAMs were ultimately classified into four modules based on the balance between error rate and stability, with each of these modules being characterized by particular marker genes and phenotypes (Supplementary Material 1, Figure S1, 2A-2B, Supplementary Material 2, Table S5). These modules included the proliferative (MKI67, TOP2A, and TUBB), lipid metabolism (APOA2, APOC2, and APOE), interferon (GBP1, IL1B, and PTPRC), and proinflammatory modules (CD63, C1QA, and C1QB). The expression levels of these modules were then used to assign TAMs into different clusters (Fig. 2C, D, Supplementary Material 2, Table S6).

SCENIC analyses additionally revealed significant differences in transcription factor expression profiles across these four subtypes of TAMs (Fig. 2E), further supporting the heterogeneous nature of TAMs within the HCC microenvironment. The Monocle 2 algorithm was further used to implement pseudotime analyses assessing the relationships between modules and cellular evolution, revealing multiple TAM developmental patterns and differentiation directions (Fig. 2F, G). These included two major expression patterns designated C1 (gradual increase) and C2 (gradual decrease) (Fig. 2H,Supplementary Material 2, Table S7). Of note, the lipid metabolism module genes APOA2 and APOE, the inflammatory module genes CD63, C1QA, and C1QB, and the interferon module gene GBP1 all presented with C1 patterns. Genes associated with the proliferation module were stably expressed across the course of TAM evolution, whereas inflammatory module genes tended to gradually rise in expression levels with TAM evolution (Fig. 2I).

### Interactions of TAM subtypes with the TME and cancer cells

**Fig. 3:**
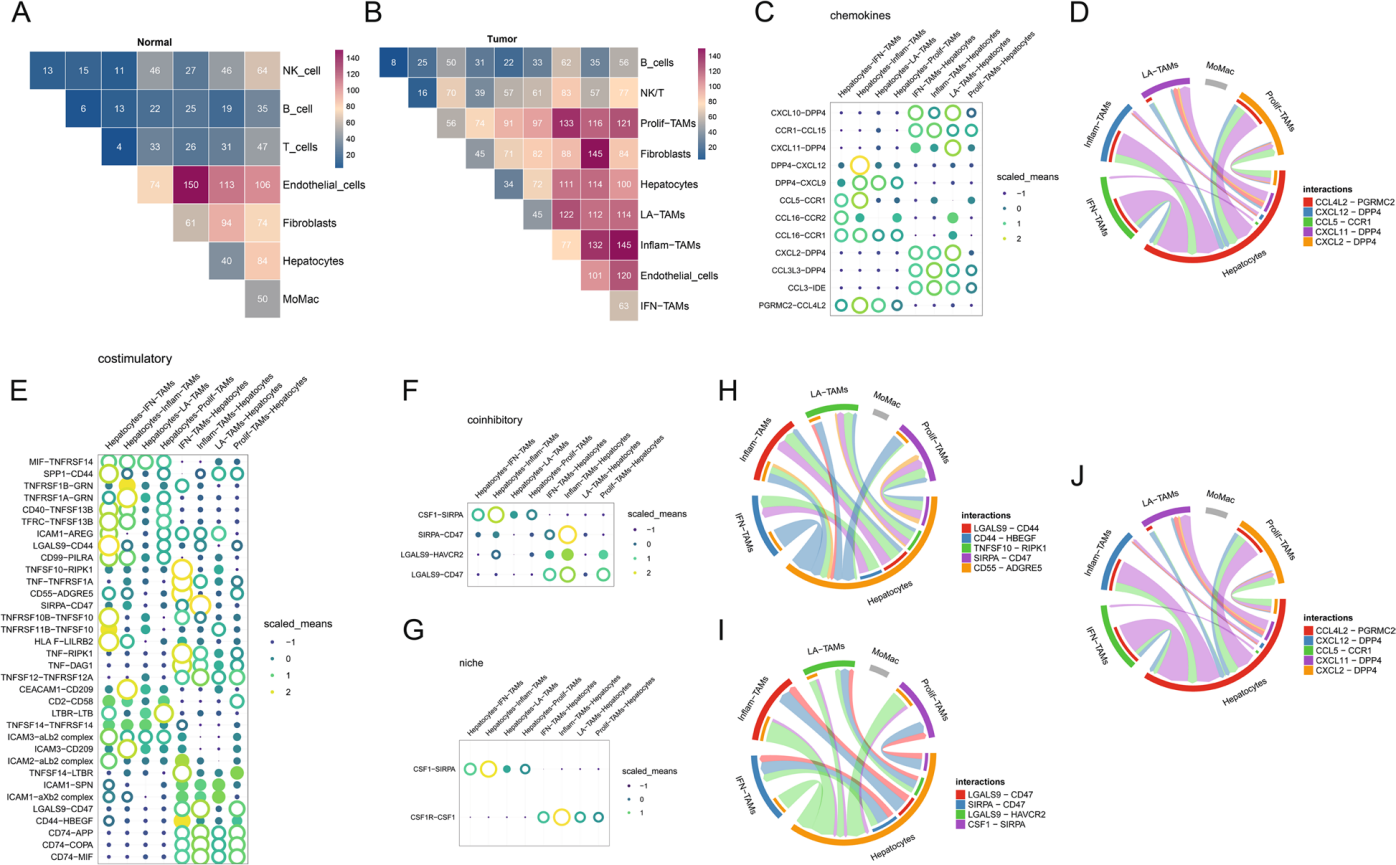
Dynamic Interactions between Subtypes of TAMs and Components of the Microenvironment. **A**, **B **Heatmap Illustrating the Number of Potential Ligand‒Receptor Pairs between Macrophages and Components of the Liver/TME, as Predicted by CellphoneDB. **C**-**J **Provide a Detailed Description of the Interaction between Macrophage Subtypes and Cancer Cells, and Highlight the Top 5 Ligand‒Receptor Pairs with the Highest Number of Interaction Relationships

TAMs can interact with other cells in the TME such that they function as vital regulators of tumor progression. To better characterize these interactions in this experimental context, CellPhoneDB was utilized, revealing a significantly increased number of total interactions between TAMs and tumor cells as well as other immune cells within tumor tissue samples as compared to healthy control tissues (Fig. 3A, B).

TAMs can express an array of chemokines, cytokines, effector molecules, and surface proteins capable of suppressing or augmenting HCC-related immune responses. The recruitment of these cells and their activation are the results of particular chemokines released within the immune-inflammatory microenvironment, ultimately triggering the polarized differentiation of TAMs into subsets associated with particular pathological conditions. Of note, IFN-TAMs, Inflam-TAMs, and LA-TAMs were all found to be closely associated with specific chemokines, as these TAMs expressed CCL4L2, CXCL12, CCL5, CXCL11, and CXCL2.

TAMs can also interact with HCC tumor cells through multiple mechanisms (Fig. 3C-J). One particularly noteworthy interaction is that between signal regulatory protein alpha (SIRPα), which is an inhibitory receptor protein that is primarily expressed by Inflam-TAMs, and CD47, which is expressed by tumor cells. IFN-TAMs primarily exhibit tumor necrosis factor (TNF) expression and can interact with a range of tumor cell receptors to modulate cellular functionality. Macrophage activity and differentiation are also closely related to the ability of colony-stimulating factor-1 (CSF-1) to signal through its receptor CSF-1R. In this study, tumor cells were found to express CSF-1, highlighting a strong link between these cells and Inflam-TAMs. These results thus support the existence of bidirectional regulatory links between Inflam-TAMs and cancer cells.

### Macrophage-derived CD63 in HCC is highly expressed in tumor tissue and is associated with a poor prognosis

**Fig. 4:**
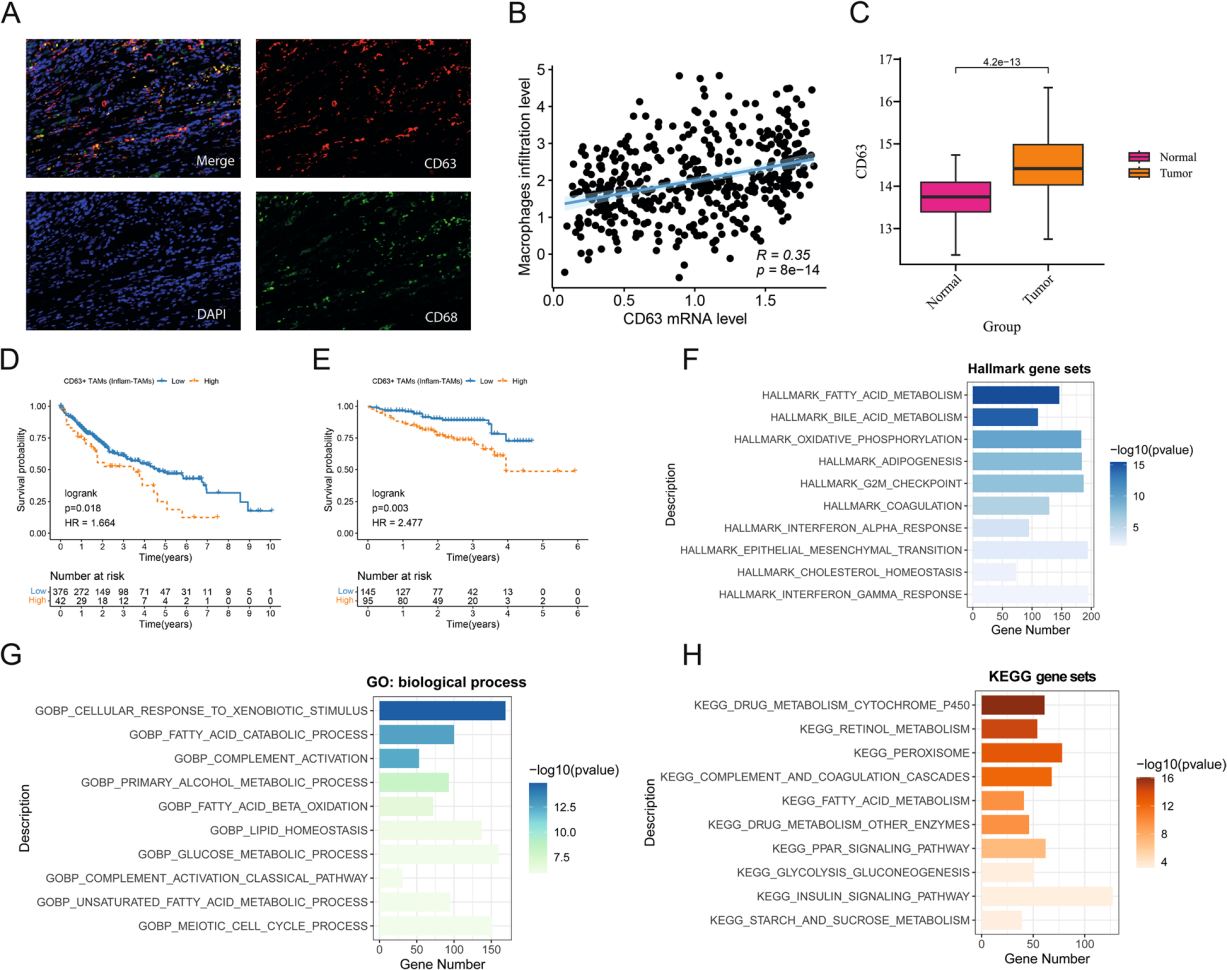
The upregulated expression of CD63 is closely related to TAMs infiltration and poor prognosis in HCC. **A **Multiplex immunohistochemistry showed that CD63 was expressed in macrophages (CD68) of hepatocellular carcinoma. **B **Correlation analysis of macrophages infiltration level and CD63 mRNA level in the TCGA-LIHC dataset. **C **Expression of CD63 mRNA in normal tissues and HCC tissues in the TCGA-LIHC dataset. **D**, **E **Kaplan‒Meier curves of overall survival (OS) of patients with different CD63 expression levels in the TCGA-LIHC and ICGC-LIHC-JP datasets. **F–H **Barplots showing the results of the functional enrichment analysis of patients with high CD63 expression in the MSigDB database hallmark, GO biological process and KEGG gene sets

As inflammation-related TAMs appear to play a role in the progression of HCC, the contributions of the inflammatory TAM marker gene CD63 to HCC development were next explored at length. To that end, multicolor immunohistochemical staining was used to assess CD63 and CD68 expression within HCC tissues. This approach revealed consistent CD63 expression by macrophages (Fig. 4A). Moreover, analyses of data from the TCGA-LIHC cohort revealed that CD63 expression was significantly positively correlated with macrophage infiltration (Fig. 4B). Pronounced D63 upregulation was also observed in tumors relative to nontumor tissues (Fig. 4C).

To establish the effects of CD63 + TAMs on prognostic outcomes for patients and the role in HCC, survival analyses were performed for two large cohorts of HCC patients. In our analysis, CD63 + TAMs (inflammation) were strongly associated with poorer prognosis in HCC patients (Fig. 4D, E, Supplementary Material 1, Figure S2).

As a means of understanding the functional relevance of the expression of CD63 in HCC, functional enrichment analyses of patients expressing high CD63 levels were additionally conducted with the MSigDB database. This approach revealed a number of biological processes enriched in the group with high CD63 expression, including the lipid metabolism, EMT, and cell cycle regulation pathways (Fig. 4F-H). Lipid homeostasis, lipid synthesis, fatty acid metabolism, and cholesterol metabolism were also all highly represented in several gene sets.

### Silencing TAM CD63 expression impairs HCC cell proliferative, migratory, and invasive activity in vitro

**Fig. 5:**
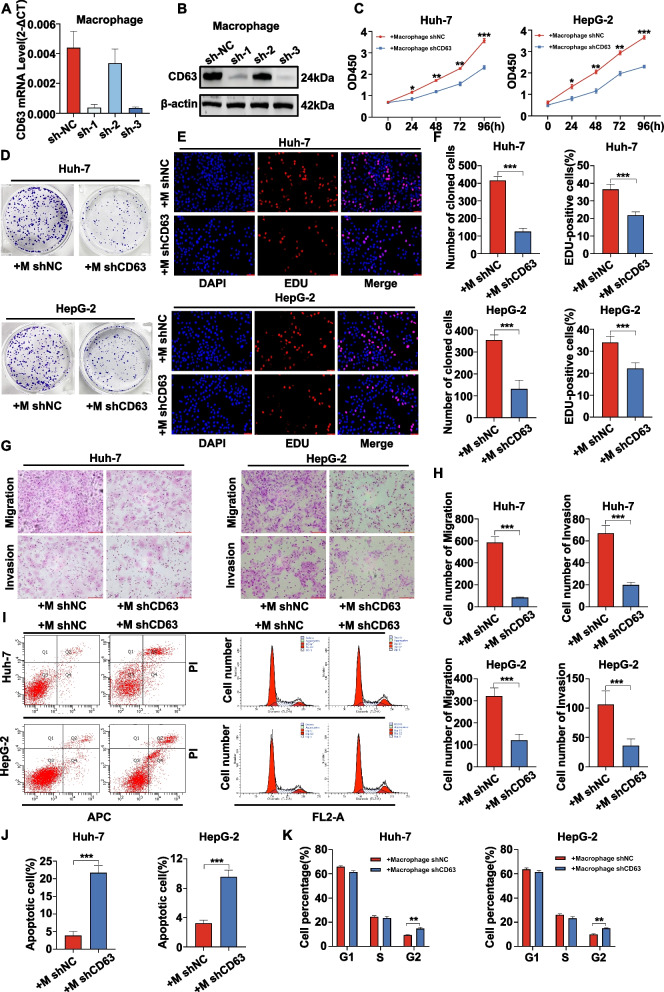
Silencing TAM CD63 expression impairs HCC cell proliferative, migratory, and invasive activity in vitro*.***A**, **B **CD63 shRNA was transfected into THP-1-derived macrophages, and the expression levels of CD63 mRNA and protein were detected using qRT‒PCR and western blotting, respectively. **C**-**E **The proliferation ability of HCC cells (Huh-7 and HepG2) was assessed using CCK8, colony formation and EdU assays after coculturing them with CD63 knockdown macrophages for 48 h. **F **Statistical charts for cloning and EdU assays. **G **The migration and invasion ability of HCC cells (Huh-7 and HepG2) were assessed after coculturing them with CD63 knockdown macrophages. **H **Statistical charts for transwell assay. **I**: The apoptosis rate and cell cycle of HCC cells (Huh-7 and HepG2) were determined by flow cytometry after transfecting macrophages with either shNC or shCD63. **J**, **K **Statistical charts for the apoptosis rate and cell cycle of HCC cells (Huh-7 and HepG2)

In an effort to understand how CD63 expression by macrophages affects HCC cell phenotypes, a series of in vitro assays was next conducted. Initially, THP-1 cells were differentiated using PMA to produce macrophages, and lentiviral vectors encoding a CD63-specific shRNA were then used to generate macrophages in which CD63 had been stably knocked down. Knockdown efficiency of CD63 was confirmed via qRT-PCR and western blotting (Fig. 5A, B). The results showed that the sh-3 interference sequence had the highest efficiency in knocking down CD63 and was determined to be applied in subsequent experiments. CCK8, clone formation and EdU assays revealed that Huh7 and HepG2 cell proliferation were significantly reduced when macrophages in which CD63 had been knocked down were present (Fig. 5C-E). The statistical charts for cloning and EdU assays are shown in Figure F. In order to further evaluate the impact of CD63 on cancer cells, transwell assays were applied to detect the migratory and invasive potential of these HCC cells. Migration and invasion capability were also significantly impaired upon co-culture with these CD63-knockdown macrophages (Fig. 5G). The statistical charts for transwell assay were shown in Fig. 5H. Flow cytometry also revealed increased Huh7 and HepG2 apoptosis following CD63 knockdown in macrophages, and cell cycle assays revealed that HCC cell numbers in the G2/M phase were significantly higher in the presence of these CD63-knockdown macrophages (Fig. 5I, K).

### CD63-overexpressing TAMs drive HCC cell proliferative, migratory, and invasive activity in vitro

**Fig. 6:**
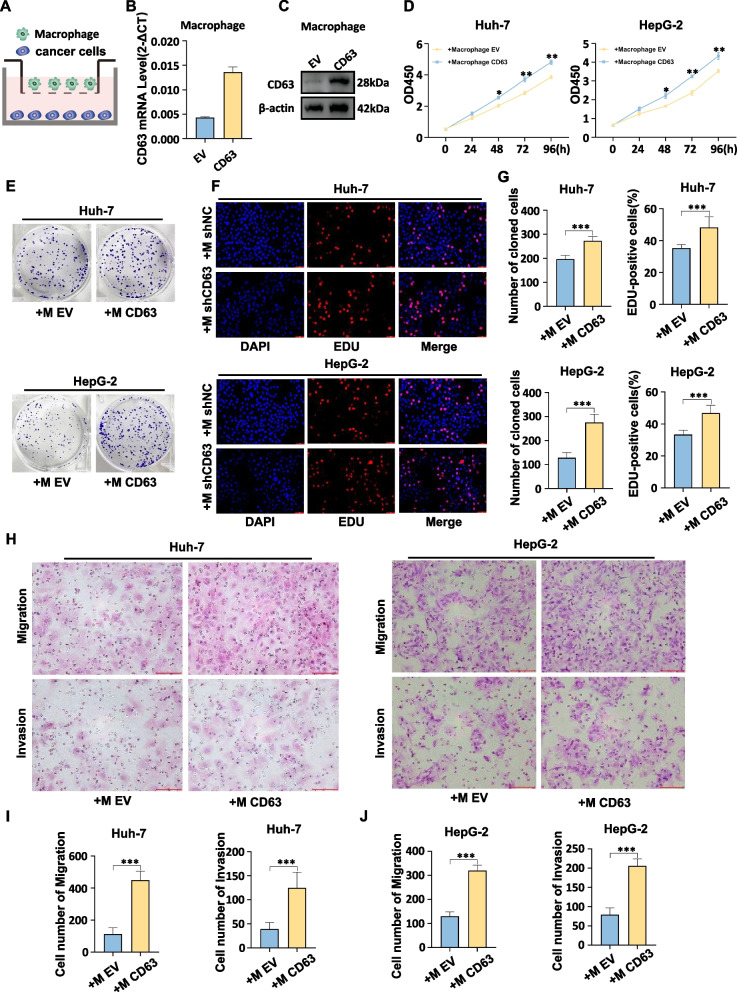
CD63-overexpressing TAMs drive HCC cell proliferative, migratory, and invasive activity in vitro*.***A **Schematic illustration of the coculture of HCC cells (Huh-7 and HepG2) and THP-1-derived macrophages. **B**, **C **THP-1 cells were differentiated into macrophages and then transiently transfected with plasmids containing CD63 cDNA or vector only. The expression of CD63 mRNA and protein was detected using qRT‒PCR and Western blot, respectively. **D**-**F **The proliferation ability of HCC cells (Huh-7 and HepG2) was assessed using CCK8, colony formation, and EdU assays after co-culturing them with macrophages transfected with CD63 vector and control vector for 48 h. **G **Statistical charts for cloning and EdU assays. **H **The migration and invasion ability of HCC cells (HepG2 and Huh-7) was examined after co-culturing them with macrophages transfected with CD63 vector and control vector. **I**, **J **Statistical charts for transwell assay

Subsequently, these CD63-overexpressing macrophages were then cultured together with either Huh7 or HepG2 HCC cells in transwell assay system for 48 h, after which HCC cells were subjected to various functional assays (Fig. 6A).

To better confirm the pro-tumor effects of macrophage CD63 expression on the growth of HCC cells, plasmids were used to stabilize the overexpression of CD63 in macrophage cells, similarly, as confirmed by qRT-PCR and western blotting (Fig. 6B, C). In line with the knockdown experiment, co-culturing CD63-overexpressing macrophages with HCC cells enhanced the proliferation of tumor cells via CCK8, clone formation and Edu assays (Fig. 6D-F). The statistical charts for cloning and EdU assays were shown in Fig. 6G. Transwell assay showed that co-culturing CD63-overexpressing macrophages with HCC cells promoted migratory and invasive potential of HCC cells (Fig. 6H-J).

### CD63 expression in TAMs can influence EMT and intracellular lipid levels in HCC cells

**Fig. 7:**
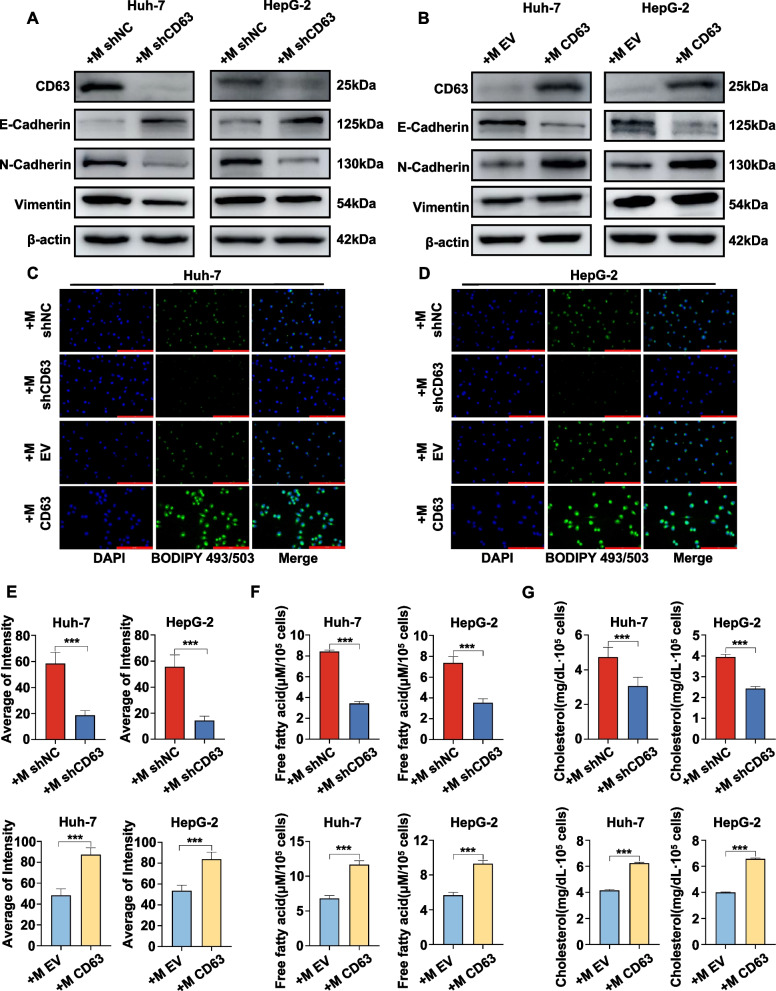
TAM-derived CD63 expression can influence EMT and intracellular lipid levels in HCC cells. **A**, **B **The EMT status of HCC cells was assessed by co-culturing them with macrophages that either had CD63 knocked down or overexpressed for a period of 48 h. **C**, **D **Representative images of BODIPY 493/503 staining (green) were captured to evaluate the presence of neutral lipids in the Huh-7 and HepG2 cells in the respective CD63 knockdown and overexpression groups. Cell nuclei were counterstained with 4’,6-diamidino-2-phenylindole (DAPI, blue). **E **Quantitative results of BODIPY 493/503. **F**, **G **The cellular contents of free fatty acids and cholesterol were quantified in Huh-7 and HepG2 cells in the CD63 knockdown and overexpression groups, respectively

To explore how the expression of CD63 by macrophages alters HCC cell functionality, the EMT induction and lipid metabolism activities were selected as targets for further study. To that end, EMT-related epithelial and mesenchymal marker proteins (E-cadherin, N-cadherin, and Vimentin) were analyzed in Huh7 and HepG2 cells. Knocking down CD63 in macrophages significantly increased the expression level of E-cadherin and reduced the expression levels of N-cadherin and Vimentin. On the other hand, an increase in the expression of CD63 in macrophages reduced the expression level of E-cadherin and increases the expression levels of N-cadherin and Vimentin (Fig. 7A-B). These results indicated that CD63 in macrophages promotes EMT.

Lastly, the impact of TAMs CD63 expression on the metabolic activity within HCC cells was evaluated through analyses of cellular lipid content, with a particular focus on lipid droplet formation in both analyzed HCC cell lines. Strikingly, macrophages silencing CD63 were able to suppress the formation of lipid droplets in these tumor cells, whereas overexpressing CD63 had the opposite effect, driving this process (Fig. 7C-E). To confirm this link between TAMs CD63 and the lipid metabolism of HCC cells, free fatty acid and cholesterol levels in Huh7 and HepG2 cells were analyzed. In this experiment, CD63 silencing significantly reduced both free fatty acid and cholesterol levels, whereas the opposite occurred in the context of CD63 overexpression (Fig. 7F, G).

## Discussion

In the present report. scRNA-seq data were leveraged to characterize the transcriptomic landscape of TAMs in HCC. Through these analyses, novel interactions between CD63 + TAMs and HCC cells were detected, supporting the key role that this subtype of TAMs plays in HCC progression [26]. TAM infiltration is a hallmark of the immunosuppressive microenvironment generally observed in HCC, and prior reports have demonstrated the context-dependent ability of TAMs to inhibit or enhance tumor growth [27, 28]. TAM heterogeneity and the interactions between these cells and HCC cells, however, have yet to be fully characterized.

Single-cell technologies have fueled an increasingly comprehensive understanding of the heterogeneous nature of cancers at the cellular level, yielding unprecedented insight into the diverse nature of TAMs through a wealth of datasets [29]. Here, an unsupervised clustering algorithm was employed to group TAMs into four subtypes, including Prolif-TAMs, LA-TAMs, IFN-TAMs, and Inflam-TAMs. Prolif-TAMs have previously been reported in pan-cancer research, and exhibit expression patterns characterized by the proliferation marker Ki-67 and several other genes associated with the cell cycle [30]. Prolif-TAMs play a role in rapid intratumoral TAM accumulation and are linked to limited differentiation, immunosuppressive phenotypes, and poorer patient prognostic outcomes [31–33]. LA-TAMs exhibit lipid-related gene-based expression signatures enriched for factors involved in the oxidative phosphorylation and lipid metabolism pathways. These cells engage a variety of mechanisms to aid in the maintenance of tissue-level metabolic homeostasis [34–36]. IFN-TAMs are M1-like macrophages in terms of their patterns of gene expression, yet they primarily function in an immunosuppressive manner that includes the degradation of tryptophan and the ability to recruit immunosuppressive regulatory T cells (Tregs) [37–39]. Inflam-TAMs present with characteristic patterns of inflammatory cytokine expression and help recruit and regulate immune cells in the context of tumor-associated inflammation [40]. HCC is a cancer that is generally associated with inflammatory activity, with chronic inflammation playing a role in HCC development [41, 42]. While the gene expression profiles of each of these TAM subtypes are distinct and they engage in different interactions with HCC cells, the Inflam-TAM subtype was particularly strongly associated with these tumor cells, suggesting that it may be particularly important as a driver of disease progression.

In the present analyses, CD63 was identified as a marker gene associated with the inflammatory HCC-related TAM expression program. CD63 was the first transmembrane tetraprotein to be identified, and it is encoded on chromosome 12q13 in the human genome. CD63 has previously been associated with the modulation of the behaviors of solid tumors exhibiting metastatic potential [8, 43]. The role that CD63 plays in the interactions between TAMs and HCC cells, however, is not well understood. Here, high levels of intratumoral CD63 expression were observed and linked to poor prognostic outcomes. In an effort to better unveil the functional roles that CD63 + TAMs play in HCC, functional and molecular analyses were performed. Through a series of in vitro assays, TAMs expressing high levels of CD63 were found to promote HCC cell proliferation, invasivity, and metastasis.

The EMT is integral to the tumor progression process, with its induction facilitating enhanced cellular motility, dissemination, and consequent metastasis [44, 45]. Here, CD63 expression by TAMs was found to upregulate mesenchymal marker expression (N-cadherin and Vimentin) by HCC cells while suppressing epithelial E-cadherin expression. Tumor metabolic reprogramming can help optimize the utilization of resources and provide effective energy homeostasis within the TME [46]. Lipid metabolism changes can significantly contribute to the progression of HCC [47, 48]. Here, the expression of CD63 by TAMs was found to be positively correlated with levels of both cholesterol and triglycerides. These data thus highlight the role that CD63 + TAMs play in the progression of HCC, in contrast with prior reports suggesting that CD63 can negatively regulate HCC [49]. The discrepancy between our findings and previous studies regarding the role of CD63 in HCC underscores the complexity of tumor biology and the variability in CD63 function depending on its cellular context. This difference could be attributed to the distinct molecular and cellular environments between macrophages within the tumor microenvironment and the cancer cells, indicating that CD63 may play dual roles in HCC progression depending on its cellular localization and the specific intercellular interactions it mediates.

## Conclusions

In conclusion, our study highlights the potential role of CD63 + TAMs in regulating HCC progression and opens up new possibilities for targeted therapy. Recognizing CD63 + TAMs as crucial regulators of HCC progression offer opportunities for therapeutic intervention. Targeting CD63 + TAMs could disrupt the pre-tumorigenic communication between TAMs and HCC cells, leading to the development of novel immunotherapeutic strategies. Additionally, the use of CD63 as a prognostic marker may help guide personalized treatment approaches for HCC patients.

### Supplementary Information

Supplementary Material 1.

Supplementary Material 2.

Supplementary Material 3.

Supplementary Material 4.

Supplementary Material 5.

## Data Availability

All date underlying this article are available in the article and in its online supplementary material. The data supporting this study's findings are available on request from the corresponding author.

## Abbreviations

TAMs: Tumor-associated macrophages
HCC: Hepatocellular carcinoma
EMT: Epithelial-mesenchymal transition
TME: Tumor microenvironment
MMPs: Matrix metalloproteinases
scRNAseq: Single-cell RNA sequencing
TCGA: The cancer genome atlas
ICGC: International cancer genome consortium
HVGs: Highly variable genes
PCA: Principal component analysis
UMAP: Uniform manifold approximation and projection
DEGs: Differentially expressed genes
GO: Gene ontology
KEGG: Kyoto encyclopedia of genes and genomes
cNMF: Consensus nonnegative matrix factorization
SCENIC: Single-cell regulatory network inference and clustering
AUC: Area under the curve
RSS: Regulon specificity score
CCK8: Cell counting Kit-8
