# Supplementary material for: CD63 + tumor-associated macrophages drive the progression of hepatocellular carcinoma through the induction of epithelial-mesenchymal transition and lipid reprogramming
Source: BMC Cancer. 2024 Jun 7;24:698. doi: 10.1186/s12885-024-12472-7 (PMC11157766; doi:10.1186/s12885-024-12472-7)
Supplement: Supplementary file 1 — Supplementary Material 1. [file 12885_2024_12472_MOESM1_ESM.docx]

Supplementary figure 1:


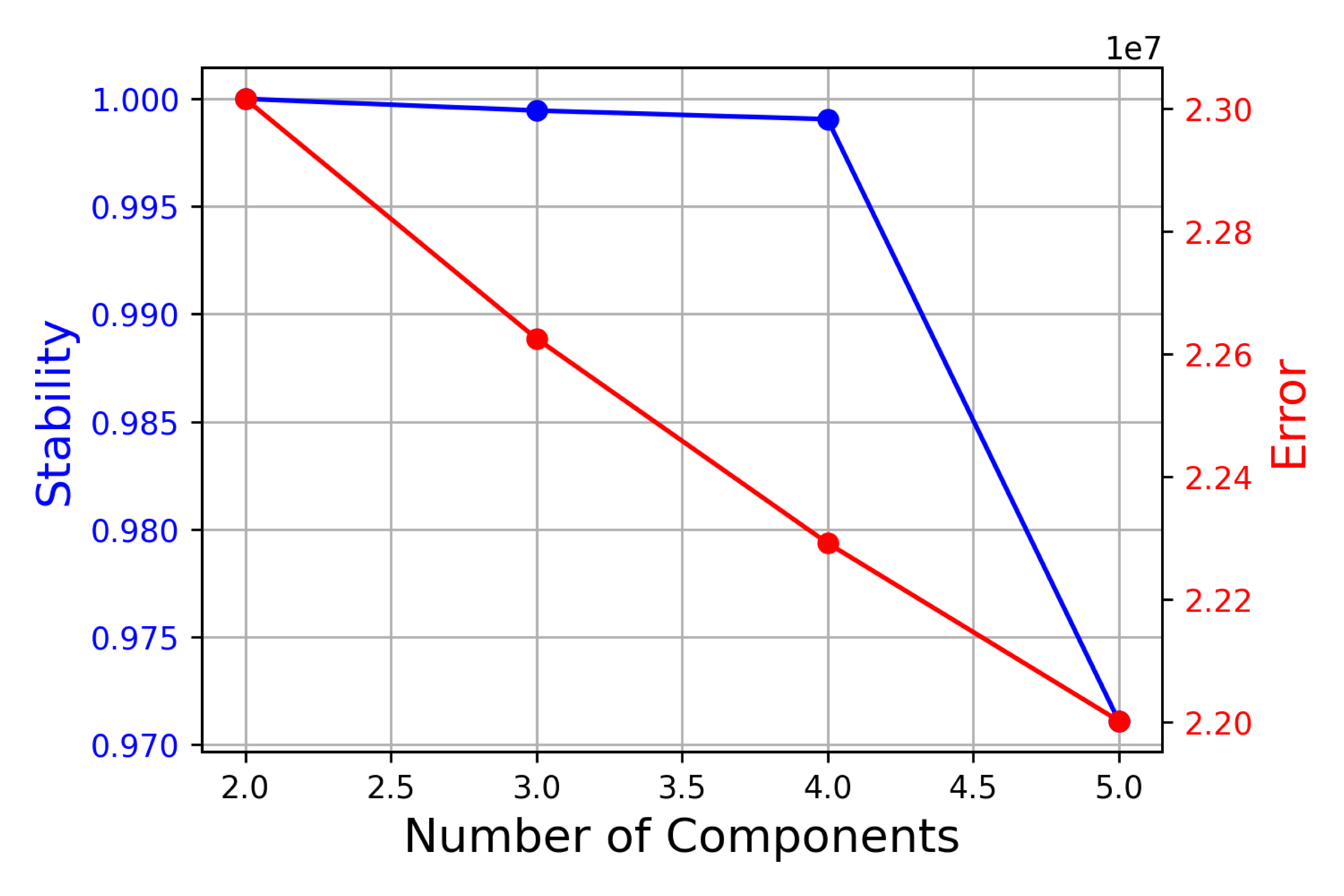


Supplementary figure1: Demographic and Quality Control Metrics of scRNA-seq Data.

Supplementary figure 2:


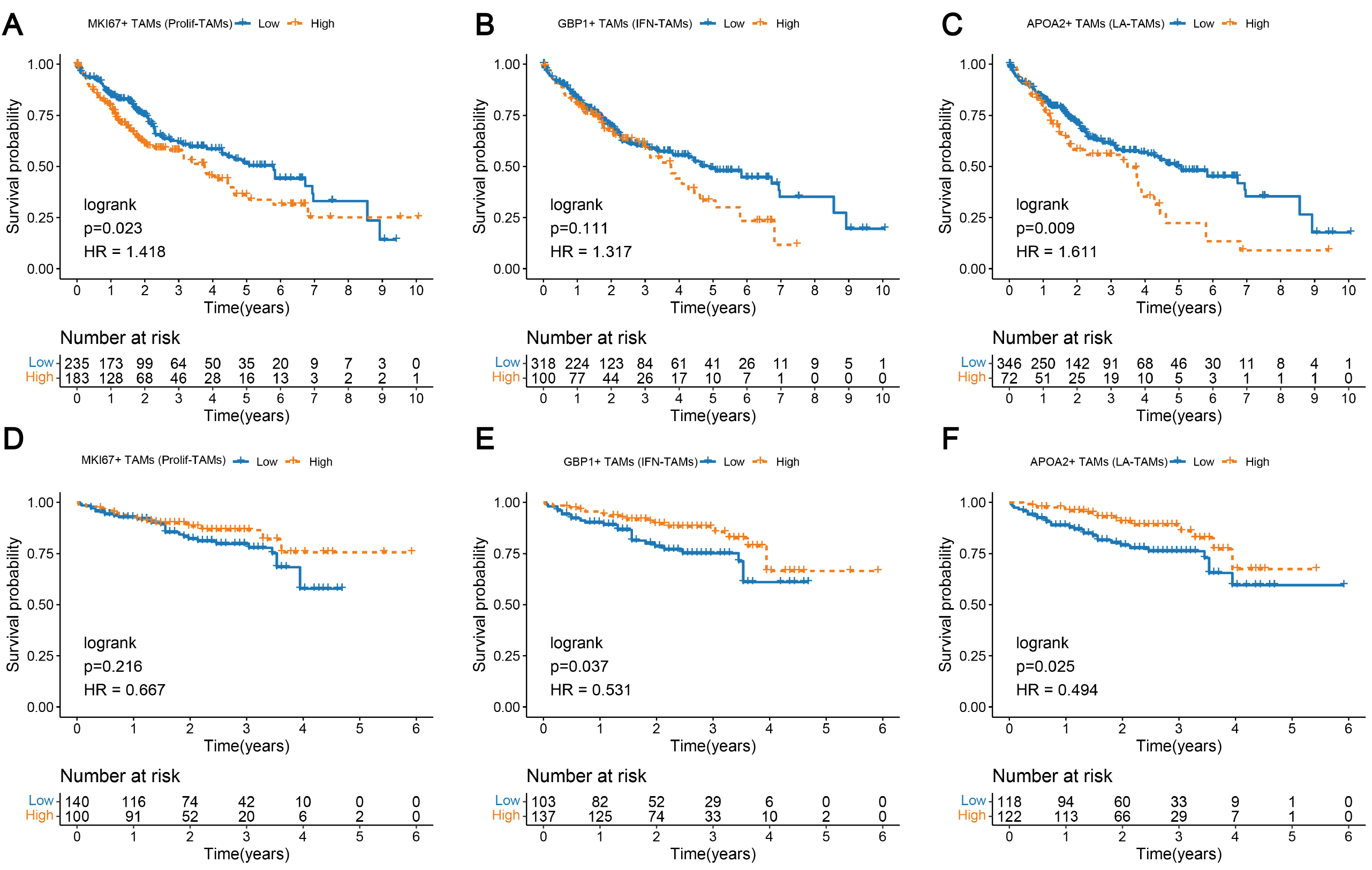


Supplementary figure 2: Survival analysis of different TAM clusters in the TCGA-LIHC and ICGC-LIRI-JP cohorts. (A-C) TCGA-LIHC cohort; (D-F) ICGC-LIRI-JP cohort.

Supplementary figure 3: Cancer cells apoptosis rate of CD63-overexpression in TAMs. (A) The apoptosis rate of Huh-7 co-cultured with macrophages transfected with EV or CD63. (B) The apoptosis rate of HepG2 co-cultured with macrophages transfected with EV or CD63.
