# Supplementary material for: CD63 + tumor-associated macrophages drive the progression of hepatocellular carcinoma through the induction of epithelial-mesenchymal transition and lipid reprogramming
Source: BMC Cancer. 2024 Jun 7;24:698. doi: 10.1186/s12885-024-12472-7 (PMC11157766; doi:10.1186/s12885-024-12472-7)
Supplement: Supplementary file 3 — Supplementary Material 3. [file 12885_2024_12472_MOESM3_ESM.pdf]

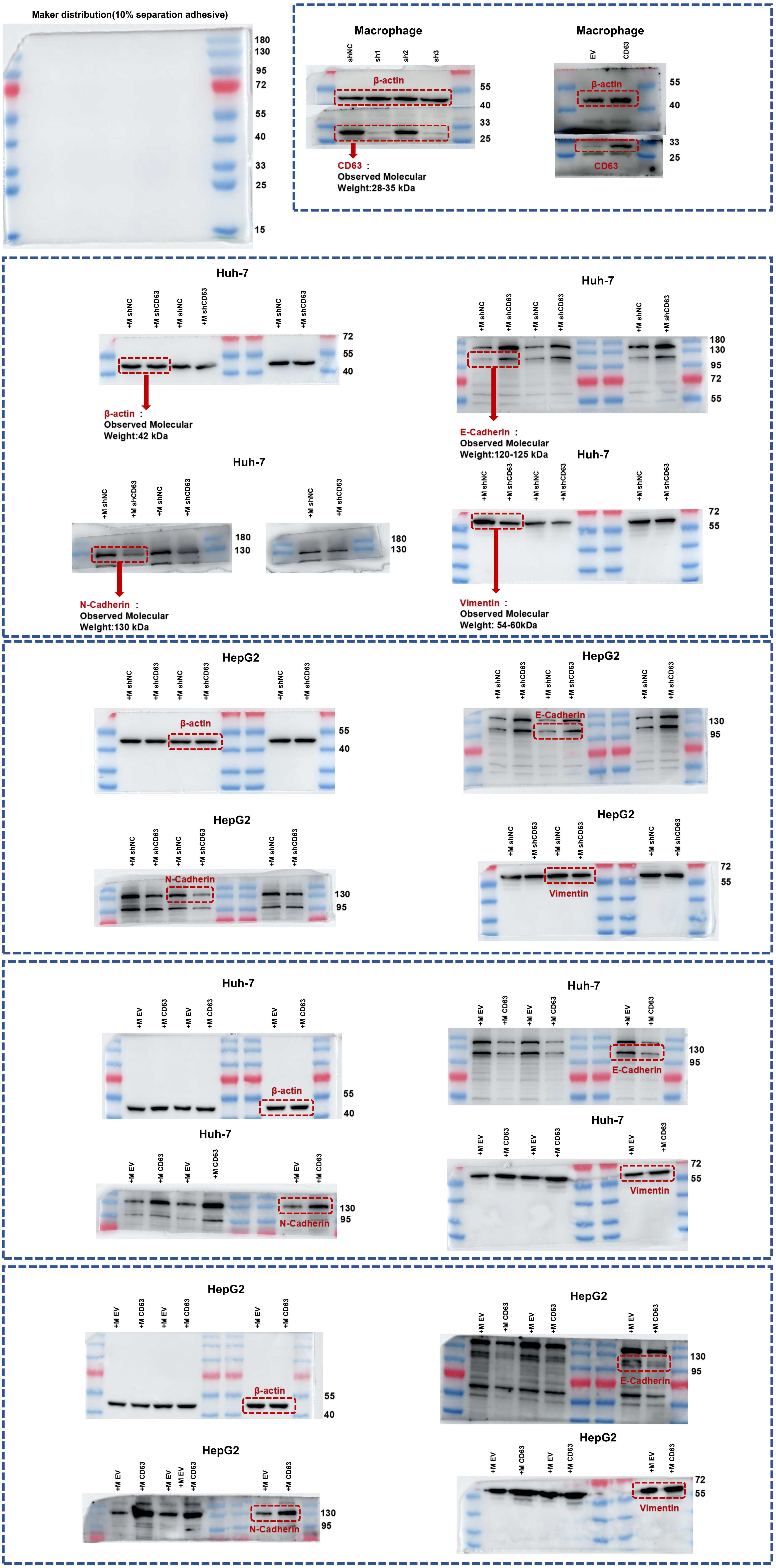

Maker distribution(10% separation adhesive)
180
130
95
72
55
40
33
25
15
Macrophage
shNC
sh1
sh2
sh3
β-actin
55
40
33
25
CD63 :
Observed Molecular
Weight:28-35 kDa
Macrophage
EV
CD63
β-actin
55
40
33
25
CD63
Huh-7
+M shNC
+M shCD63
+M shNC
+M shCD63
+M shNC
+M shCD63
72
55
40
β-actin :
Observed Molecular
Weight:42 kDa
Huh-7
+M shNC
+M shCD63
+M shNC
+M shCD63
+M shNC
+M shCD63
180
130
95
72
55
E-Cadherin :
Observed Molecular
Weight:120-125 kDa
Huh-7
+M shNC
+M shCD63
+M shNC
+M shCD63
+M shNC
+M shCD63
180
130
180
130
N-Cadherin :
Observed Molecular
Weight:130 kDa
Huh-7
+M shNC
+M shCD63
+M shNC
+M shCD63
+M shNC
+M shCD63
72
55
Vimentin :
Observed Molecular
Weight: 54-60kDa
HepG2
+M shNC
+M shCD63
+M shNC
+M shCD63
+M shNC
+M shCD63
β-actin
55
40
HepG2
+M shNC
+M shCD63
+M shNC
+M shCD63
+M shNC
+M shCD63
E-Cadherin
130
95
HepG2
+M shNC
+M shCD63
+M shNC
+M shCD63
+M shNC
+M shCD63
N-Cadherin
130
95
HepG2
+M shNC
+M shCD63
+M shNC
+M shCD63
+M shNC
+M shCD63
72
55
Vimentin
Huh-7
+M EV
+M CD63
+M EV
+M CD63
+M EV
+M CD63
β-actin
55
40
Huh-7
+M EV
+M CD63
+M EV
+M CD63
+M EV
+M CD63
130
95
E-Cadherin
Huh-7
+M EV
+M CD63
+M EV
+M CD63
+M EV
+M CD63
130
95
N-Cadherin
Huh-7
+M EV
+M CD63
+M EV
+M CD63
+M EV
+M CD63
72
55
Vimentin
HepG2
+M EV
+M CD63
+M EV
+M CD63
+M EV
+M CD63
β-actin
55
40
HepG2
+M EV
+M CD63
+M EV
+M CD63
+M EV
+M CD63
130
95
E-Cadherin
HepG2
+M EV
+M CD63
+M EV
+M EV
+M CD63
+M EV
+M CD63
130
95
N-Cadherin
HepG2
+M EV
+M CD63
+M EV
+M CD63
+M EV
+M CD63
72
55
Vimentin
