# Supplementary material for: CD63 + tumor-associated macrophages drive the progression of hepatocellular carcinoma through the induction of epithelial-mesenchymal transition and lipid reprogramming
Source: BMC Cancer. 2024 Jun 7;24:698. doi: 10.1186/s12885-024-12472-7 (PMC11157766; doi:10.1186/s12885-024-12472-7)
Supplement: Supplementary file 4 — Supplementary Material 4. [file 12885_2024_12472_MOESM4_ESM.pdf]

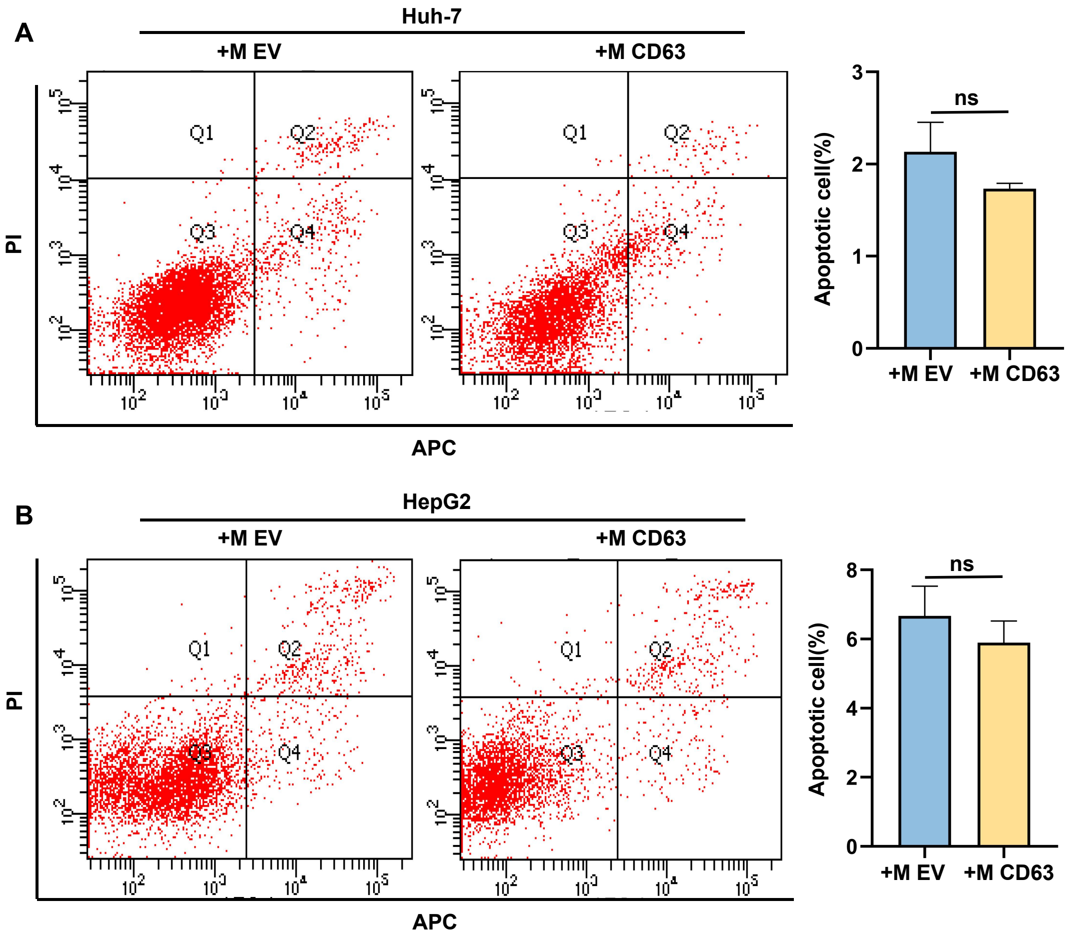

Cancer cells apoptosis rate of CD63-overexpression in TAMs.
(A) The apoptosis rate of Huh-7 co-cultured with macrophages transfected with EV or CD63.
(B) The apoptosis rate of HepG2 co-cultured with macrophages transfected with EV or CD63.
